# Simulation test for impartment of use-dependent plasticity by inactivation of axonal potassium channels on hippocampal mossy fibers

**DOI:** 10.3389/fncel.2023.1154910

**Published:** 2023-04-26

**Authors:** Fumeng Zheng, Haruyuki Kamiya

**Affiliations:** Department of Neurobiology, Hokkaido University Graduate School of Medicine, Sapporo, Japan

**Keywords:** axon, hippocampus, inactivation, mossy fiber, potassium channel, simulation

## Abstract

Modification of axonal excitability directly impacts information transfer through the neuronal networks in the brain. However, the functional significance of modulation of axonal excitability by the preceding neuronal activity largely remains elusive. One remarkable exception is the activity-dependent broadening of action potential (AP) propagating along the hippocampal mossy fibers. The duration of AP is progressively prolonged during repetitive stimuli and facilitated presynaptic Ca^2+^ entry and subsequent transmitter release. As an underlying mechanism, accumulated inactivation of axonal K^+^ channels during AP train has been postulated. As the inactivation of axonal K^+^ channels proceeds on a timescale of several tens of milliseconds slower than the millisecond scale of AP, the contribution of K^+^ channel inactivation in AP broadening needs to be tested and evaluated quantitatively. Using the computer simulation approach, this study aimed to explore the effects of the removal of the inactivation process of axonal K^+^ channels in the simple but sufficiently realistic model of hippocampal mossy fibers and found that the use-dependent AP broadening was completely abolished in the model replaced with non-inactivating K^+^ channels. The results demonstrated the critical roles of K^+^ channel inactivation in the activity-dependent regulation of axonal excitability during repetitive action potentials, which critically imparts additional mechanisms for robust use-dependent short-term plasticity characteristics for this particular synapse.

## Introduction

Use-dependent modification of the synaptic strength offers the neural basis for encoding temporal information of the activity into the subsequent synaptic strength. The gain and the time course of the use-dependent modification are defined strictly depending on the types of synapses for their roles in neural computation. The hippocampal mossy fiber synapse is well-known for exhibiting characteristic robust short-term synaptic plasticity with large frequency-dependent facilitation over a more than 10-fold range (Salin et al., 1996; Henze et al., 2000; Nicoll and Schmitz, 2005). The underlying mechanisms have been studied extensively using experimental approaches, including direct subcellular recordings from the axon terminals (Bischofberger et al., 2006; Schmidt-Hieber et al., 2008; Ohura and Kamiya, 2018; Vandael et al., 2021) and fluorescent Ca^2+^ measurement (Kamiya and Ozawa, 1999; Kamiya et al., 2002). In addition to the classical residual calcium mechanism (Katz and Miledi, 1968; Zucker and Regehr, 2002), it has been demonstrated that modification of the duration of axonal action potentials contributes substantially to robust short-term plasticity at this synapse (Geiger and Jonas, 2000). Activity-dependent broadening of axonal action potentials has been attributed to the accumulated inactivation of axonal K^+^ channels different from those observed in squid giant axons which do not inactivate during prolonged depolarization (Hodgkin and Huxley, 1952). As demonstrated for pituitary nerve terminals (Jackson et al., 1991) and invertebrate neurons (Byrne and Kandel, 1996), the previous study using direct subcellular recording from the mossy fiber terminals showed that the axonal K^+^ currents display robust inactivation during prolonged depolarization with a time constant of several tens of milliseconds (Geiger and Jonas, 2000; see also Cooper et al., 1998). However, as each action potential during the train lasts for a millisecond timescale, it is less certain whether these axonal K^+^ channels inactivate during repetitive action potentials with a millisecond time course. To get insight into the roles of inactivating K^+^ channels at the mossy fiber bouton, we attempted to test for the consequence of replacing the K^+^ channel model with those without inactivation as a classical Hodgkin–Huxley type model using a computational approach. For quantitative evaluation of the contribution of accumulated inactivation of axonal K^+^ channels during repetitive action potentials, we compared the simulation results between those obtained by the models containing or lacking inactivation properties. It was found that the activity-dependent broadening of action potential observed in the mossy fiber model with inactivating K^+^ channels was completely abolished when the K^+^ channel model was replaced with a non-inactivating one. The calculated K^+^ current was progressively decreased while the calculated Ca^2+^ current was increased during the repetitive action potentials, and these use-dependent modulations were completely abolished when the K^+^ channel model was replaced with a non-inactivating one. Taken together, our simulation data suggested that inactivating profiles of K^+^ channels impart additional mechanisms for short-term synaptic plasticity of an extremely large wide dynamic range characteristic for the hippocampal mossy fiber synapse.

## Materials and methods

### Simulation

The simulated membrane potential (V_m_) was calculated according to the model suggested by Engel and Jonas (2005) based on the data recorded from mossy fiber boutons (see also Kamiya, 2019). All model and simulation files have been uploaded to the ModelDB database (https://senselab.med.yale.edu/modeldb/ accession no. 267617). The model assumed a Hodgkin–Huxley-type gating model adapted to channels recorded in mossy fiber terminals. For K^+^ channel, we used either the non-inactivating K^+^ channel from Schmidt-Hieber and Bischofberger, 2010 (hhmfb.mod, ModelDB accession no. 128079) or the inactivating K^+^ channel that they constructed by taking the non-inactivating K^+^ channel and adding an inactivation parameter based on recombinant K_V_1.4 channels described by Wissmann et al., 2003 (KIn.mod, ModelDB accession no. 128079). Simulations were performed using NEURON 7.8 for Windows (Hines and Carnevale, 1997). The passive electrical properties of the axon were assumed to be uniform, with a specific membrane capacitance Cm of 1 μF cm−2, a specific membrane resistance Rm of 10,000 Ω cm2, and an intracellular resistivity Ri of 70 Ω cm (Engel and Jonas, 2005; Alle and Geiger, 2006). The resting membrane potential was set to −80 mV. The structure of the mossy fiber (Acsády et al., 1998; Henze et al., 2000) was approximated by a soma (diameter, 10 μm), 10 axonal cylinders (diameter, 0.2 μm; length, 100 μm), and 10 *en passant* boutons (diameter, 4 μm). The number of segments was 1 μm^−1^ in all simulations. The reversal potential of the leak conductance was set to −81 mV to maintain stability. For all simulations in this study, the time step was set as 0.01 ms to describe the time course of fast action potentials and the underlying currents.

Voltage-gated Na^+^ channels, K^+^ channels, and leakage channels were inserted into the soma, axon, and boutons, as in the previous study (Kamiya, 2019). The Na^+^ conductance density was set to 50 mS cm^−2^ for the axon and boutons and 10 mS cm^−2^ for the soma. The K^+^ conductance density was set to 36 mS cm^−2^ throughout all parts of the neurons, unless otherwise stated. In some simulations, K^+^ conductance density was changed to half (18 mS cm^−2^) or 10 times (360 mS cm^−2^) from the original value of 36 mS cm^−2^ to observe the change in action potential broadening by repetitive stimulation. Action potentials were evoked by the injection of depolarizing current into the soma (2 ms, 0.2 nA). The equilibrium potentials for Na^+^ and K^+^ ions were assumed to be +50 and −85 mV, respectively. In some simulations, the non-inactivating K^+^ channel model, which lacks inactivation properties, was used instead of inactivating K^+^ channel model. The hhmfb.mod, a Hodgkin–Huxley-type model for the set of sodium, potassium, and leakage channels adapted to channels in mossy fiber terminals, was downloaded from ModelDB (accession no. 128079) for a non-inactivating I_K_ model for sodium and potassium conductances. For inactivating I_K_ model, the potassium conductance of hhmfb.mod was omitted and instead inserted Kin.mod, a Hodgkin–Huxley model of inactivating K channels representing kinetics of K_V_1.4, downloaded from the same ModelDB (accession no. 128079).

The models of presynaptic Ca^2+^ channels of P/Q type, N type, and R type are reconstructed previously (Kamiya, 2022) using the kinetic parameters obtained experimentally (Li et al., 2007). The gating models of these presynaptic Ca^2+^ channels assumed six states gating model consisting of five closed states (C0–C4) and a single open state (O) for each subtype. Transitions from C0 to C4 are assumed to be voltage-dependent, while a transition from C4 to O is voltage-independent. The equilibrium potential for Ca^2+^ ions was assumed to be +60 mV according to the value obtained from the recording from hippocampal mossy fiber terminals (Li et al., 2007) and used for the simulation in their study.

For simulations in **Figure 7**, the kmb.mod, a model for the K_V_7 M-type K channels on the mossy fiber boutons was downloaded from ModelDB (accession no. 245417) and implemented evenly to the axons and boutons. The conductance density was set to 5 mS cm^−2^ according to the simulation in the previous study (Martinello et al., 2019).

Axonal action potentials, as well as K^+^ and Ca^2+^ currents at the mossy fiber boutons evoked by the repetitive action potentials of 50 times at 10, 20, and 50 Hz, were calculated for testing the use-dependent modification.

## Results

### Simulated action potentials with inactivating and non-inactivating K^+^ channel models

**Figure 1:**
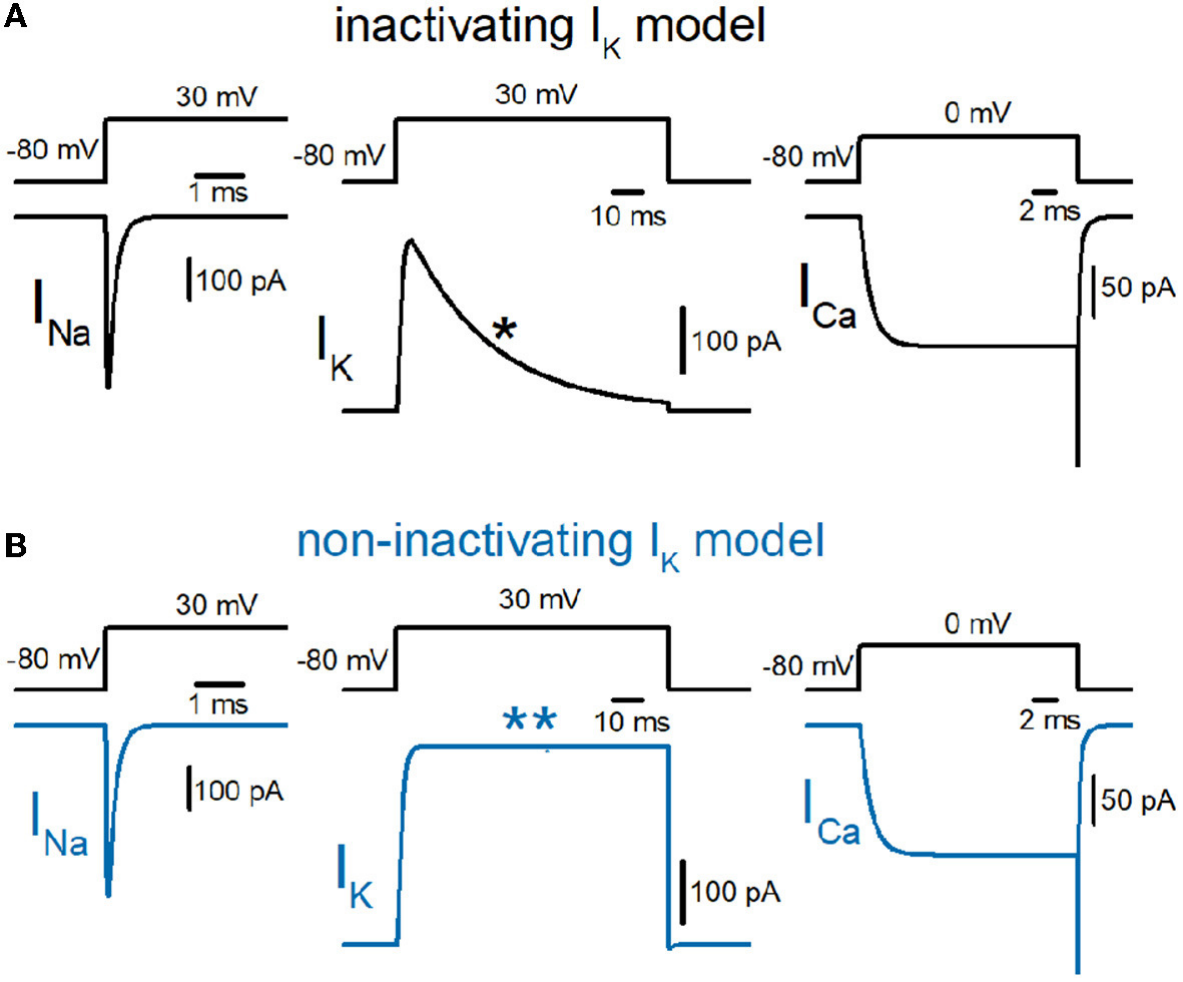
Reconstruction of axonal sodium, potassium, and presynaptic calcium current on the hippocampal mossy fibers. **(A)** Kinetic properties of sodium (I_Na_), potassium (I_K_), and calcium (I_Ca_) current of the models reconstructed from the experimental data obtained from hippocampal mossy fiber boutons. Single-compartment models of 7 μm diameter were used for these simulations. The I_K_ currents with fast inactivation (as marked by the asterisk) were used for calculation (black traces). **(B)** Those of the models whose potassium channel property was replaced with the non-inactivating one (as marked by a double-asterisk, blue traces).

**Figure 2:**
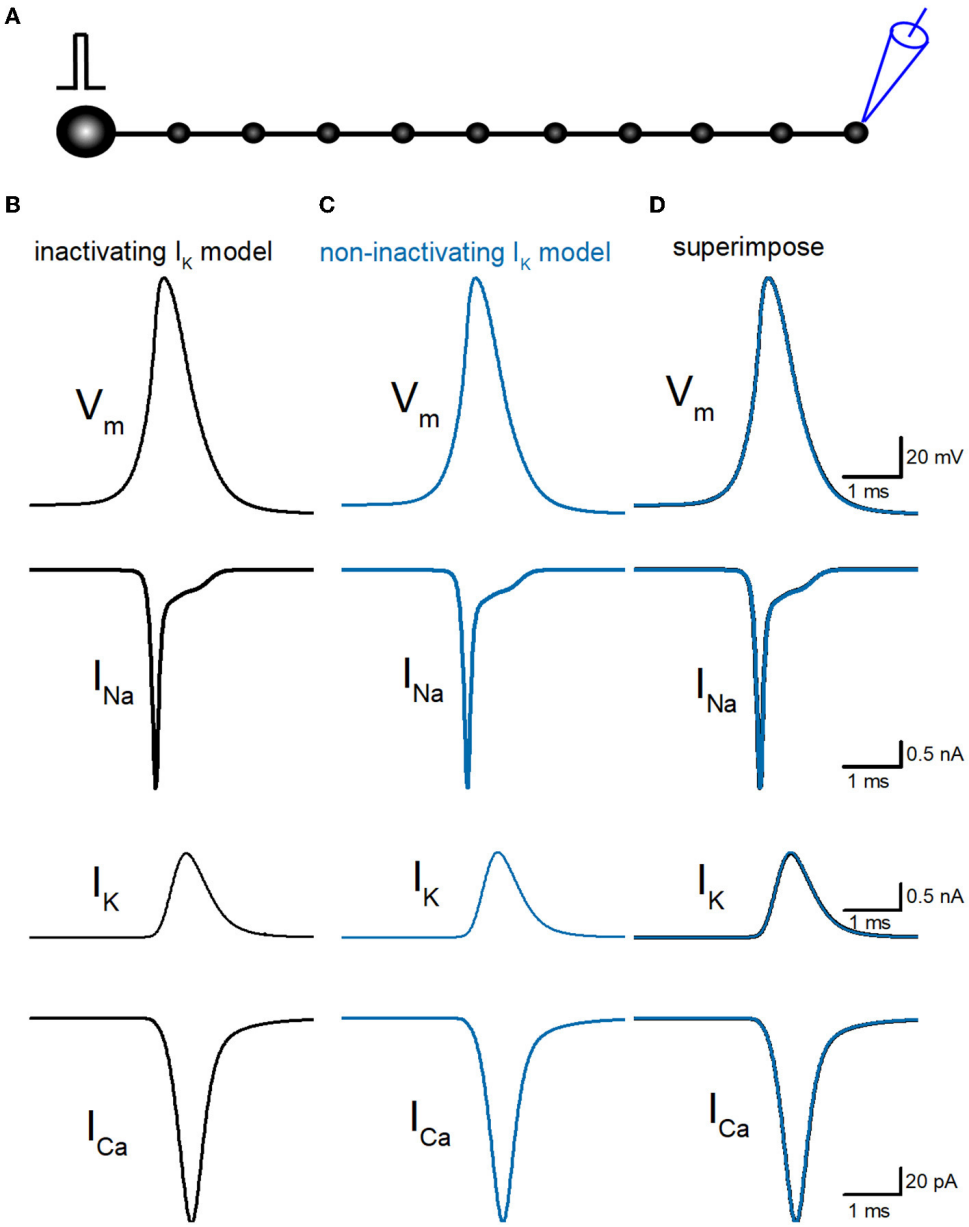
Reconstruction of propagating action potentials with inactivating and non-inactivating axonal K^+^ channels. **(A)** Schematic drawing of the multi-compartment model of the granule cell and mossy fiber was reconstructed according to the previous study by Alle and Geiger (2006). **(B)** Simulated propagating action potentials at the mossy fiber bouton (V_m_), axonal Na^+^ (I_Na_), K^+^ (I_K_), and Ca^2+^ (I_Ca_) currents during the action potentials with the inactivating I_K_ model. **(C)** Those of non-inactivating I_K_ model (blue traces). **(D)** Superimposed traces of **(B, C)**.

So far, we have reconstructed a simple and sufficiently realistic model of hippocampal mossy fibers with a “*pearl chain*” structure (Engel and Jonas, 2005) implemented with models of axonal voltage-dependent Na^+^ and K^+^ channels (Kamiya, 2019) and presynaptic Ca^2+^ channels (Kamiya, 2022) based on the kinetic parameters determined experimentally (Geiger and Jonas, 2000; Engel and Jonas, 2005; Li et al., 2007). First, we tested the time course of Na^+^ (I_Na_), K^+^ (I_K_), and Ca^2+^ (I_Ca_) currents in a single-compartment model with a 7 μm diameter. I_Na_ and I_K_ were tested with 100 ms depolarization to 30 mV, and I_Ca_ was tested with 20 ms depolarization to 0 mV. K^+^ current in the original model shows robust inactivation during 100 ms depolarization to 30 mV (Figure 1A) as shown in the black asterisk in the lower trace. In contrast, the K^+^ current in the non-inactivating I_K_ model does not show inactivation during the same depolarizing command (Figure 1B) as shown in the blue double-asterisk in the lower trace. Using these two model sets of I_K_ with and without inactivation in the multicompartment model of hippocampal mossy fibers (Figure 2A), we compared the waveform of the propagating action potentials (V_m_), as well as K^+^ (I_K_) and Ca^2+^ (I_Ca_) currents in the models equipped with the inactivating (Figure 2B) and the non-inactivating I_K_ (Figure 2C). As shown in superimposed traces, the replacement of the K^+^ channel model with the one lacking inactivation shows a minimal effect on the repolarization phase (Figure 2D).

### Use-dependent action potential broadening in hippocampal mossy fiber model

**Figure 3:**
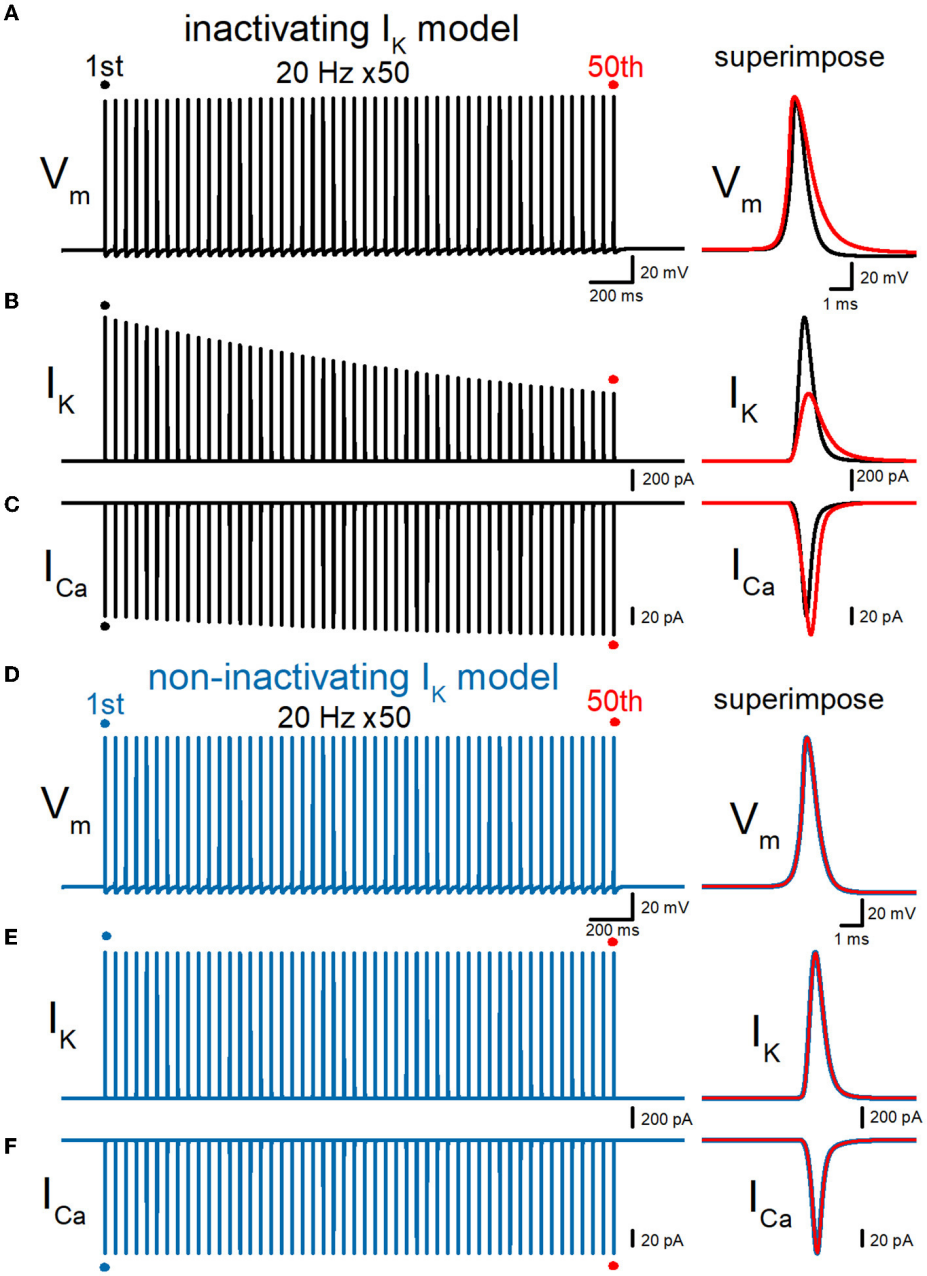
Effects of repetitive stimuli on action potentials, axonal I_K_, and presynaptic I_Ca_ simulated with inactivating and non-inactivating axonal K^+^ channels. **(A)** Simulated propagating action potentials at the mossy fiber bouton (V_m_) elicited by repetitive stimuli of 50 times at 20 Hz in the inactivating I_K_ model. **(B)** Axonal K^+^ current elicited by the repetitive action potentials (I_K_) in inactivating I_K_ model. **(C)** Ca^2+^ currents (I_Ca_) elicited by axonal action potentials during the repetitive stimuli in inactivating the I_K_ model. **(D)** Simulated propagating action potentials at the mossy fiber bouton (V_m_) elicited by repetitive stimuli of 50 times at 20 Hz in the non-inactivating I_K_ model. **(E)** Axonal K^+^ current elicited by the repetitive action potentials (I_K_) in the non-inactivating I_K_ model. **(F)** Ca^2+^ currents (I_Ca_) elicited by axonal action potentials during the repetitive stimuli in the non-inactivating I_K_ model. In each right panel, the responses to the 50th stimulus (red traces) were superimposed with that elicited by the 1st stimulus (black or blue traces) for comparison.

Then, we examined whether our model displays a broadening of action potential in response to repetitive stimuli as reported in the experimental study (Geiger and Jonas, 2000). To examine the use-dependent broadening of action potentials, 50 times stimuli at 10, 20, and 50 Hz were tested to observe the frequency dependency. In these conditions, all action potentials were elicited by the repetitive stimuli faithfully, excluding the possibility of conduction block by inactivation of Na^+^ channels. Repetitive stimuli of 50 times at 20 Hz progressively slowed the repolarization, thereby causing a broadening of action potentials (Figure 3A). Half-duration of the 50th action potential (1.18 ms) was 152% of the 1st action potential (0.777 ms) for the 20-Hz train. We also calculated K^+^ (I_K_) and Ca^2+^ (I_Ca_) currents during repetitive action potentials, to look for the consequence of action potential broadening. I_K_ during action potential progressively decreased the amplitude (Figure 3B), as expected for cumulative inactivation by the repetitive trains. The peak amplitude of the 50th I_K_ (0.732 nA) was 47.0% of the 1st responses (1.56 nA) for the 20-Hz train. On the other hand, I_Ca_ during the action potential is progressively increased by the repetitive train of action potentials (Figure 3C). The peak amplitude of the 50th I_Ca_ (174 pA) was 117% of the 1st responses (149 pA) for the 20-Hz train. The Ca^2+^ charge was also calculated as it was reported to be more correlated with AP broadening than I_Ca_ peak amplitude (Geiger and Jonas, 2000). The charge due to the inflow of Ca^2+^ was enhanced by repetitive stimuli due to the potential activity-dependent changes in the synaptic strength being more related to the physiological states affecting the signaling in the brain. The calculated charge of Ca^2+^ inflow was enhanced by 165% of control by a 20-Hz train of 50 stimuli.

### Effects of replacement with non-inactivating K^+^ channels

To quantitatively evaluate the contribution of accumulated inactivation of axonal K^+^ channels, next we attempted to replace the K^+^ channel model with those lacking inactivation. The same repetitive stimuli of 50 times at 20 Hz did not induce a broadening of action potentials (Figure 3D). Half-duration of the 50th action potential (0.773 ms) was 100% of the 1st action potential (0.772 ms) for the 20-Hz train. As a consequence, the calculated I_K_ and I_Ca_ did not show robust use-dependent changes in the amplitude (Figures 3E, F). The peak amplitude of the 50th I_K_ (1.57 nA) and I_Ca_ (149 pA) were 99.6 and 102% of the 1st responses (1.58 nA, 149 pA) for the 20-Hz train. Taking into account all these results, it was supposed that axonal K^+^ channels on the hippocampal mossy fibers undergo progressive inactivation during repetitive APs, and then cause use-dependent broadening of action potentials, which potentially contributes as an additional mechanism for short-term synaptic plasticity with an extremely wide dynamic range at this synapse.

### Frequency dependency of the use-dependent AP broadening

**Figure 4:**
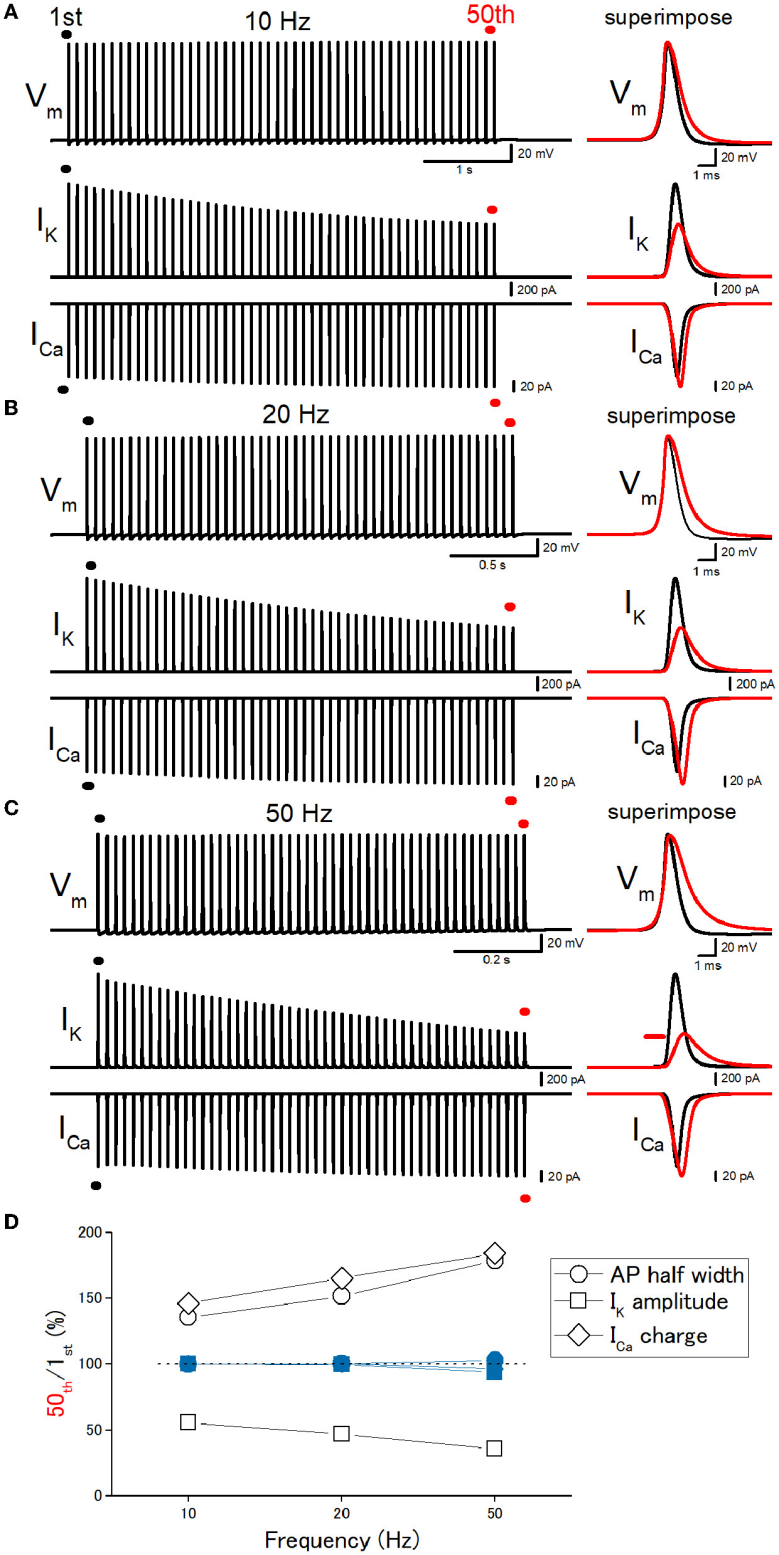
Frequency dependency of the effects of repetitive stimulation. **(A)** Simulated propagating action potentials at the mossy fiber bouton (V_m_), axonal K^+^ current (I_K_), and presynaptic Ca^2+^ currents (I_Ca_) elicited by the repetitive stimuli of 50 times at 10 Hz. **(B)** Action potentials elicited by the repetitive stimuli of 50 times at 20 Hz. **(C)** Action potentials elicited by the repetitive stimuli of 50 times at 50 Hz. In each right panel, the responses to the 50th stimulus (red traces) were superimposed with that elicited by the 1st stimulus (black traces) for comparison. **(D)** Frequency dependency of the effects. The relative values of 50th responses/1st responses of the half-width of AP (open circles), the peak amplitude of I_K_ (open squares), and the charge of I_Ca_ (open diamonds). The data of the simulation using the non-inactivating I_K_ models are similarly shown in blue symbols.

**Figure 5:**
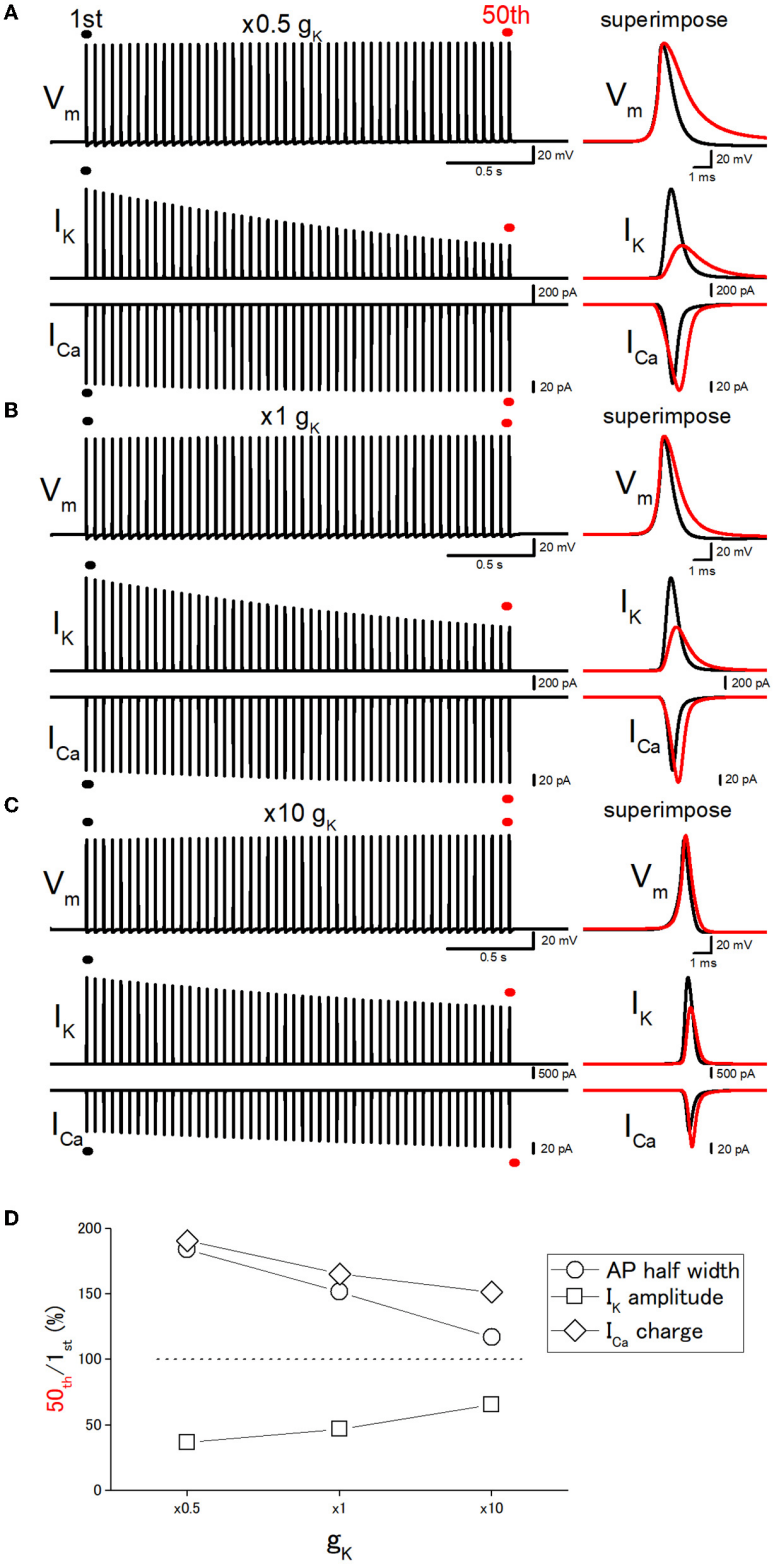
Effects of changes in the potassium conductance (g_K_) on the use-dependent broadening of axonal action potentials. **(A)** Simulated propagating action potentials at the mossy fiber bouton (V_m_), axonal K^+^ current (I_K_), and presynaptic Ca^2+^ currents (I_Ca_) elicited by the repetitive stimuli of 50 times at 20 Hz with the models in which the potassium conductance (g_K_) was reduced to half (× 0.5) of the original model. **(B)** Action potentials simulated with the original (× 1) g_K_ value. **(C)** Action potentials simulated with the model in which the g_K_ value was increased to 10 times the original one. In each right panel, the responses to the 50th stimulus (red traces) were superimposed with that elicited by the 1st stimulus (black traces) for comparison. **(D)** Dose-dependent relationships of the effects of changes in g_K_ values. The relative values of 50th responses/1st responses of the half-width of AP (open circles), the peak amplitude of I_K_ (open squares), and the charge of I_Ca_ (open diamonds).

Then, we explored the frequency dependency of the effects of repetitive stimulation. A train of 50 stimuli at 10 Hz caused a broadening of action potentials in the inactivating I_K_ model (Figure 4A). Half-duration of the 50th action potential (1.05 ms) was 136% of the 1st action potential (0.777 ms) for the 10-Hz train (open circles, Figure 4D). Half-duration of the 50th action potential (1.18 ms) was 152% of the 1st action potential (0.777 ms) for the 20-Hz train (Figures 4B, D). Half-duration of the 50th action potential (1.39 ms) was 179% of the 1st action potential (0.777 ms) for the 50-Hz train (Figures 4C, D). I_K_ during action potential progressively decreased the amplitude (Figure 4D, open squares), as expected for cumulative inactivation by the repetitive trains. I_Ca_ showed the use-dependent changes in the amplitude in a frequency-dependent manner (Figure 4D, open diamonds). The peak amplitudes of the 50th I_K_, I_Ca_, and the charge of I_Ca_ were 55.5, 114, and 146% of the 1st responses for the 10-Hz train (Figures 4A, D). The peak amplitudes of the 50th I_K_ and I_Ca_ were 47.0, 117, and 165% of the 1st responses (1.56 nA, 150 pA) for the 20-Hz train (Figures 4B, D). The peak amplitudes of the 50th I_K_, I_Ca_, and the charge of I_Ca_ were 35.6, 112, and 184% of the 1st responses (1.56 nA, 150 pA) for the 50-Hz train (Figures 4C, D). These effects were exclusively observed in the simulation with the inactivating I_K_ model and never observed in the simulation with the non-inactivating I_K_ model (blue-filled symbols in Figure 5D). All the results indicate that use-dependent broadening of axonal action potentials propagating along mossy fibers, possibly due to cumulative inactivation of axonal K^+^ channels. Robust changes in the amplitudes of I_Ca_ suggest that this mechanism substantially contributes to the large-amplitude short-term synaptic plasticity characteristic for this particular synapse.

### Effect of changes in g_*K*_ on the use-dependent AP broadening

Next, it was attempted to test whether the changes in the basal duration of the action potential affect the activity-dependent broadening, as expected from the scenario that cumulative inactivation of I_K_ during the repetitive action potentials governs the broadening of action potentials. For this purpose, the g_K_ (potassium conductance) values were changed systematically. The 10 times increase in g_K_ level (from 36 to 360 mS cm^−2^) shortened the half-duration of action potential from 0.777 ms to 0.523 ms by speeding up the repolarization phase as shown in Figure 5C. It should be noted that repetitive stimuli of 50 times at 20 Hz induced a smaller broadening of action potentials when g_K_ level was increased 10 times. With 10 times g_K_ of 360 mS cm^−2^, the half-duration of the 50th action potential, the amplitudes of I_K_ and I_Ca_, and the charge of I_Ca_ were 116, 65.6, 138, and 151% of the 1st action potential for the 20-Hz train (Figures 5C, D), while the half-duration of the 50th action potential (1.18 ms), the amplitudes of I_K_ and I_Ca_, and the charge of I_Ca_ was 152, 47.0, 117 165% of the 1st action potential (0.777 ms) with the original gK of 36 mS cm^−2^ (Figures 5B, D). On the other hand, when the gK value was decreased to half (18 mS cm^−2^) of the original value of 36 mS cm^−2^, the half-duration of the 50th action potential, the amplitudes of I_K_ and I_Ca_, and the charge of I_Ca_ were 184, 36.6, 109, and 191% of the 1st action potential (0.904 ms) for the 20-Hz train (Figures 5A, D). All the results are consistent with the notion that the duration of the action potential is critical for the use-dependent broadening of action potentials because cumulative inactivation of I_K_ during the repetitive action potentials is expected to occur strongly, therefore resulting in the larger broadening of action potentials.

### Simulation of the responses at the *en passant* bouton

**Figure 6:**
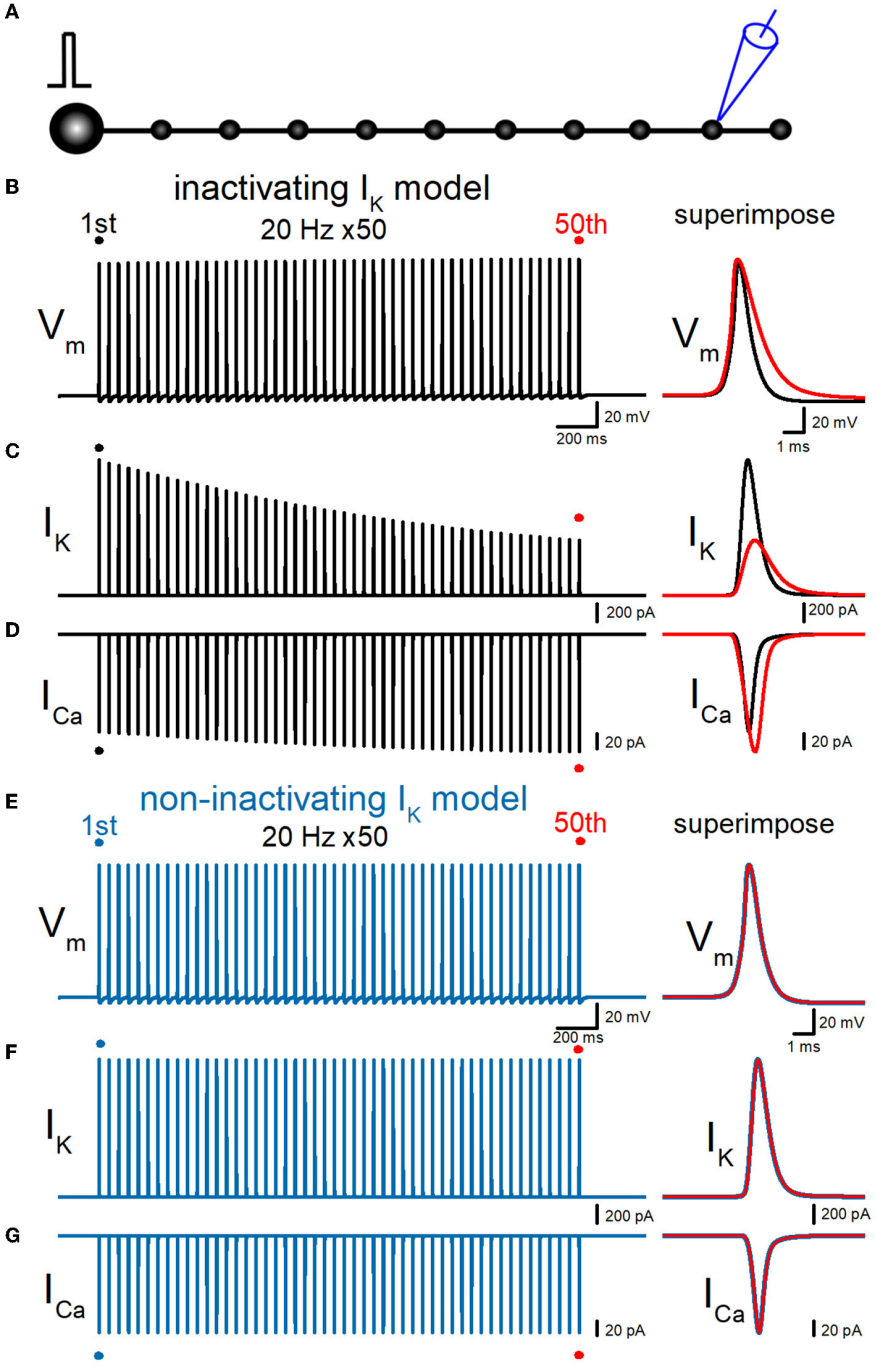
Effects of repetitive stimuli on action potentials, axonal I_K_, and presynaptic I_Ca_ recorded from the *en passant* boutons. **(A)** Schematic drawing of the multi-compartment model of the granule cell and mossy fiber and the recording site at the ninth *en passant* boutons. **(B)** Simulated propagating action potentials at the mossy fiber bouton (V_m_) elicited by repetitive stimuli of 50 times at 20 Hz in the inactivating I_K_ model. **(C)** Axonal K^+^ current elicited by the repetitive action potentials (I_K_) in inactivating I_K_ model. **(D)** Ca^2+^ currents (I_Ca_) elicited by axonal action potentials during the repetitive stimuli in inactivating the I_K_ model. **(E)** Simulated propagating action potentials at the mossy fiber bouton (V_m_) elicited by repetitive stimuli of 50 times at 20 Hz in the non-inactivating I_K_ model. **(F)** Axonal K^+^ current elicited by the repetitive action potentials (I_K_) in the non-inactivating I_K_ model. **(G)** Ca^2+^ currents (I_Ca_) elicited by axonal action potentials during the repetitive stimuli in the non-inactivating I_K_ model. In each right panel, the responses to the 50th stimulus (red traces) were superimposed with that elicited by the 1st stimulus (black or blue traces) for comparison.

So far, simulations were made to calculate the responses from the bouton at the end of the axon as illustrated schematically in Figure 2A, for comparison with the experimental findings obtained from giant boutons. This may raise the concern that the recording from the bouton on the end of the axon may have to take into account the sealed-end effects. To address this issue, we repeated the simulations to calculate the responses from the ninth *en passant* boutons as illustrated in Figure 6A. Similar to the recording from the boutons at the end of the axon in Figure 3, repetitive stimuli of 50 times at 20 Hz progressively slowed the repolarization and thereby caused a broadening of action potentials. Half-duration of the 50th action potential (1.47 ms) was 165% of the 1st action potential (0.890 ms) for the 20-Hz train (Figure 6B). I_K_ during action potential progressively decreased the amplitude (Figure 6C). The peak amplitude of the 50th I_K_ (0.629 nA) was 40.6% of the 1st responses (1.55 nA) for the 20-Hz train. On the other hand, I_Ca_ during the action potential is progressively increased by the repetitive train of action potentials (Figure 6D). The peak amplitude of the 50th I_Ca_ (165 pA) was 121% of the 1st responses (136 pA) for the 20-Hz train. The calculated Ca^2+^ charge was enhanced by 192% of control by a 20-Hz train of 50 stimuli. Thus, similar activity-dependent modulation of action potential half-width, I_K_ amplitude, and I_Ca_ charges was reproduced at the *en passant* bouton. Importantly, these activity-dependent changes were almost abolished when the axonal K^+^ channel model was replaced with a non-inactivating K^+^ channel model (Figures 6E–G). All the results in the series of simulations support the conclusion that accumulated inactivation of axonal K^+^ channels underlies activity-dependent broadenings of APs.

### Effect of implementing K_*V*_7 M-type K^+^ channel model

**Figure 7:**
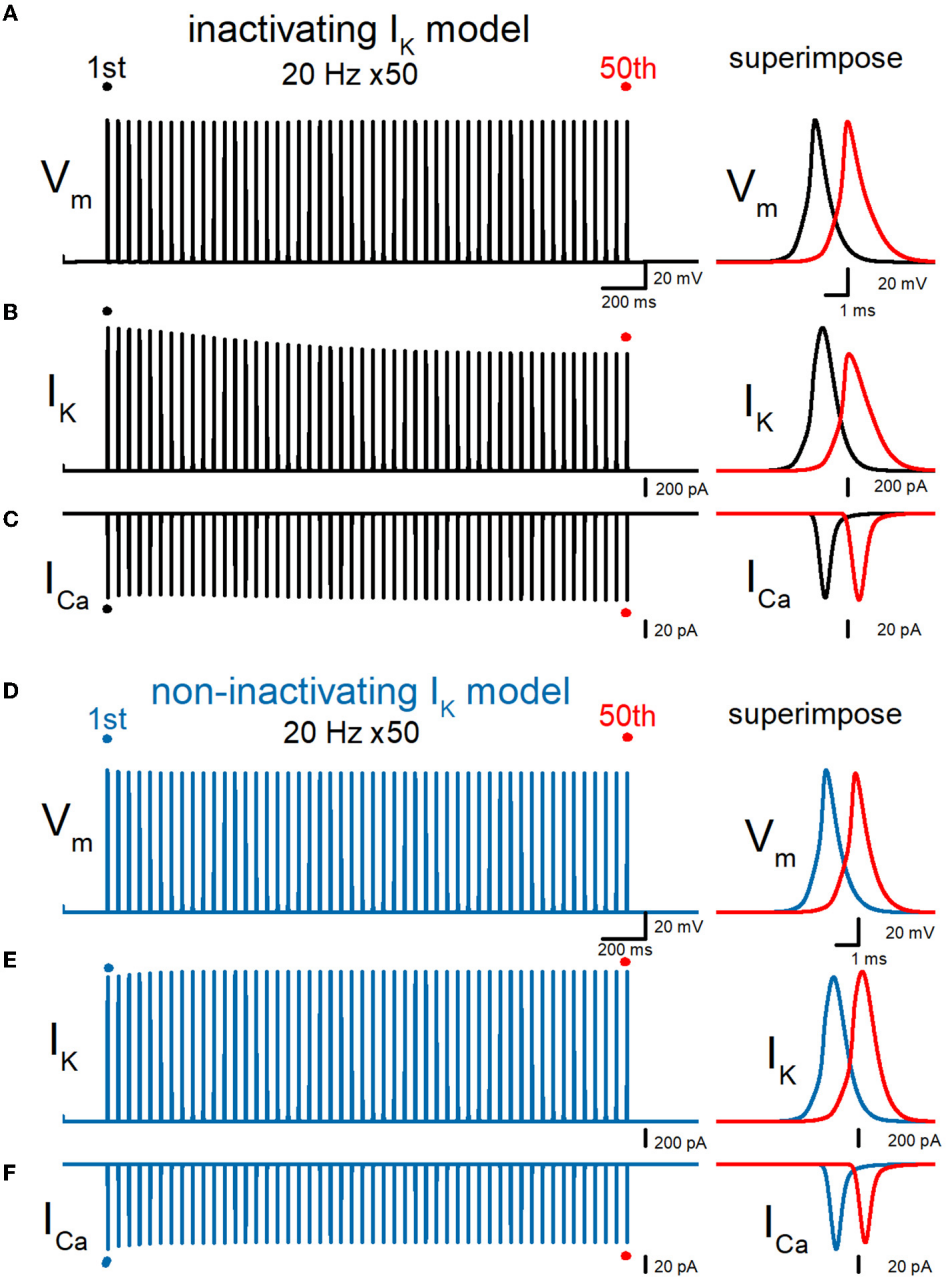
Effects of repetitive stimuli on action potentials, axonal I_K_, and presynaptic I_Ca_ with axonal K_V_7 M-type K channels. **(A)** Simulated propagating action potentials at the mossy fiber bouton (V_m_) elicited by repetitive stimuli of 50 times at 20 Hz in the inactivating I_K_ model implemented with the K_V_7 M-type K channel model. **(B)** Axonal K^+^ current elicited by the repetitive action potentials (I_K_) in inactivating I_K_ model. **(C)** Ca^2+^ currents (I_Ca_) elicited by axonal action potentials during the repetitive stimuli in inactivating the I_K_ model. **(D)** Simulated propagating action potentials at the mossy fiber bouton (V_m_) elicited by repetitive stimuli of 50 times at 20 Hz in the non-inactivating I_K_ model. **(E)** Axonal K^+^ current elicited by the repetitive action potentials (I_K_) in the non-inactivating I_K_ model. **(F)** Ca^2+^ currents (I_Ca_) elicited by axonal action potentials during the repetitive stimuli in the non-inactivating I_K_ model. In each right panel, the responses to the 50th stimulus (red traces) were superimposed with that elicited by the 1st stimulus (black or blue traces) for comparison.

All the previous simulation was tested by the simple model of mossy fibers implemented with voltage-dependent Na^+^-, K^+^-, and Ca^2+^ channels models reconstructed from the experimentally recorded properties of the channels from the mossy fiber boutons. As previous experiments have demonstrated the presence of K_V_7 (Martinello et al., 2019), K_V_3, and Ca-dependent K^+^ channels (Alle et al., 2011) using direct subcellular recording, the information is limited and has not constructed the detailed channel models for quantitative simulation. To examine the effect of K_V_7 M-type K^+^ channels, we repeated the simulations with the model implemented with the model used in the previous study (Martinello et al., 2019) and tested the effect of repetitive stimuli 50 times at 20 Hz. It was noted that the peak of action potentials was delayed for the 50th action potentials as shown in the right superimposed traces, expecting from the shunting due to these channels being active at the resting membrane potentials. The repetitive stimuli slowed the repolarization and caused a broadening of action potentials. Half-duration of the 50th action potential (1.08 ms) was 120% of the 1st action potential (0.896 ms) for the 20-Hz train (Figure 7A). I_K_ during action potential progressively decreased the amplitude (Figure 7B). The peak amplitude of the 50th I_K_ (1.48 nA) was 81.5% of the 1st responses (1.81 nA) for the 20-Hz train. On the other hand, I_Ca_ during the action potential is progressively increased by the repetitive train of action potentials (Figure 7C). The peak amplitude of the 50th I_Ca_ (112 pA) was 102% of the 1st responses (110 pA) for the 20-Hz train. The calculated Ca^2+^ charge was enhanced by 118% of control by a 20-Hz train of 50 stimuli. Thus, similar activity-dependent modulation of AP half-width, I_K_ amplitude, and I_Ca_ charges was reproduced at the *en passant* bouton. Importantly, these activity-dependent changes were almost abolished when the axonal K^+^ channel model was replaced with a non-inactivating K^+^ channel model (Figures 7D–F). It is obvious to need revision of the model to be more realistic by supplementing with the properties and exact localization of these channels on the mossy fiber axons by the precise experimental approach in future studies.

## Discussion

In this study, a numerical simulation approach was adopted to examine the contribution of the inactivation of potassium channels in an activity-dependent broadening of action potentials propagating on the hippocampal mossy fibers. Using a simple but sufficiently realistic model of hippocampal mossy fiber supplemented with inactivating and non-inactivating K^+^ channels on axonal membranes, we compared the duration of action potentials during repetitive stimuli and found that use-dependent broadening of action potentials occurs with the model implemented with inactivating K^+^ channels, while the model with non-inactivating K^+^ channels does not show the broadening of action potentials. All the findings are supporting the notion that the inactivation property of the axonal K^+^ channels imparts use-dependent modification of the synaptic strength in addition to the well-established residual Ca^2+^ mechanism for short-term synaptic plasticity.

### Inactivating K^+^ channels on the hippocampal mossy fiber axon

Recent advances in subcellular recordings from the axons have updated several classical views on physiological understandings of axons in the central nervous system. One remarkable notion different from those obtained by classical studies is the inactivation property of axonal potassium channels. For instance, K^+^ channels on the hippocampal mossy fiber terminals were demonstrated to display robust fast inactivation during prolonged depolarization (Geiger and Jonas, 2000; Alle et al., 2011) as expected from the kinetic properties of channels composed of K_V_1 and K_V_3 subunits, clearly different from the non-inactivating property of Hodgkin–Huxley-type potassium channels in squid giant axons (Hodgkin and Huxley, 1952). This notion is not specific to this particular type of synapse, as the voltage-dependent K^+^ current recorded from the calyx of Held, the best-characterized axon terminals in the central nervous system, also displays fast inactivation upon prolonged depolarization (Dodson et al., 2002; Ishikawa et al., 2003; Tong et al., 2010; Choudhury et al., 2020). Direct axonal recording study from cerebellar stellate interneuron revealed that inactivating K_V_1 and K_V_3 subunits differently distributed along the course of axons (Rowan et al., 2014, 2016) also supported the notion that inactivating K^+^ channels critically involved in the fine-tuning of axonal signaling (Johnston et al., 2010).

Inactivating properties are supposed to be involved in “analog-digital facilitation” representing the enhancement of synaptic transmitter release by the prolonged depolarization of axonal membrane potentials (Debanne et al., 2011, 2013; Ohura and Kamiya, 2016). Slowing of action potential repolarization caused by prolonged membrane depolarization modulates transmitter release from the axon terminals of layer V pyramidal neurons in the cerebral cortex (Shu et al., 2006). Similar analog modulation has been demonstrated in the axons of cultured CA3 pyramidal neurons (Sasaki et al., 2011) and the calyx of Held (Richardson et al., 2022). As action potentials propagating along mossy fibers were followed by substantial after depolarization lasting for several tens of ms (Geiger and Jonas, 2000), it may also affect the waveforms of subsequent action potentials during repetitive action potentials.

As another consequence of the inactivation of axonal K^+^ channels, use-dependent broadening of axonal action potentials due to slowed repolarization has been suggested experimentally (Geiger and Jonas, 2000), although awaiting evaluation of quantitative consistency. As I_K_ critically determines the duration of axonal action potentials at the hippocampal mossy fiber boutons (Alle et al., 2009), a slight change in the axonal I_K_ would impact the subsequent synaptic transmission. This study attempted to test this notion by computer simulation using a model of a hippocampal mossy fiber axon. Consistent with the notion, a model implemented with inactivating K^+^ channels displayed notable action potential broadening by repetitive stimuli. In contrast, the replacement of the model with non-inactivating K^+^ channels did not change the duration of action potentials by repetitive action potentials. These results demonstrated the roles of K^+^ channel inactivation in a use-dependent broadening of action potentials.

In the previous experimental study, the authors tested the cumulative inactivation of K^+^ channels by using a repetitive voltage steps protocol (Geiger and Jonas, 2000). The K^+^ current elicited by +20 mv voltage pulses for 3 ms from the −90 mV holding potential repeating at 100 Hz (7 ms interpulse intervals) progressively decreased during the repetitive voltage pulses. However, action potentials recorded at the mossy fiber terminals were much briefer than 3 ms (half-duration 379 ± 8 μs) and therefore need to be evaluated quantitatively using action potential waveform. This study demonstrated that the K^+^ current elicited by action potentials progressively declines during repetitive trains, supporting the notion of cumulative inactivation of K^+^ channels by repetitive action potentials.

### Inactivating axonal K^+^ channel imparts use-dependent short-term plasticity

Supposing a brief depolarization during action potentials inactivates a fraction of axonal K^+^ channels, it is expected that a use-dependent decrease in K^+^ current occurs in a frequency-dependent manner. Consistent with the prediction, we confirmed the cumulative frequency-dependent effects on action potential-driven K^+^ current. Likewise, simulated presynaptic Ca^2+^ current was also enhanced frequency-dependently. In our previous study (Kamiya, 2022), the models of P/Q type, N-type, and R-type Ca^2+^ channels were reconstructed by adopting kinetic parameters obtained by the voltage-clamp experiments in direct recordings from the mossy fiber terminals (Li et al., 2007). Using these Ca^2+^ channel models, Ca^2+^ currents during action potentials were calculated and found to progressively increase the amplitude by the repetitive stimuli, as expected from the broadening of action potentials. This implicates the inactivation of axonal K^+^ channels imparts use-dependent short-term synaptic plasticity at hippocampal mossy fiber synapse within the range of physiological frequency.

It should be noted that the duration of the simulated action potentials in this study (half-duration of 0.773 ms) was longer than that of experimentally observed action potentials (half-duration of 0.379 ms) as reported previously (Geiger and Jonas, 2000). One of the possible reasons for the slower action potentials in our simulation is the difference in temperature between the simulation and the experiments. Engel and Jonas (2005) measured sodium current at room temperature and described the kinetic parameters of gating of Na^+^ channels on the mossy fiber terminals, and the model adopted in this study used these parameters. On the other hand, action potentials were recorded at a physiological temperature of 34°C and thus would expect to speed up the action potential time course (Geiger and Jonas). Although the models need to be improved by taking into account the temperature, the simulated action potentials in this study seem similar to the simulated action potentials reported previously (Engel and Jonas, 2005) and therefore used for the analysis of the contribution of K^+^ channel inactivation in use-dependent action potential broadening.

In this study, a series of numerical simulations using a simple model of hippocampal mossy fiber was performed to test the possible contribution of K^+^ channel inactivation in activity-dependent modification of axonal excitability. The model implemented with inactivating K^+^ channels displays robust use-dependent broadening of action potentials by repetitive stimuli, while that with non-inactivating K^+^ channels does not change the duration. Our simulations also demonstrated the frequency dependency of the effect as expected from the cumulative nature of the inactivation of axonal K^+^ channels. The enhanced Ca^2+^ entry by repetitive action potentials was also demonstrated quantitatively. Taking all pieces of evidence, the inactivating property of axonal action potentials provides an important component for activity-dependent short-term synaptic plasticity with an extremely wide dynamic range at the hippocampal mossy fiber synapse.

## Data availability statement

The raw data supporting the conclusions of this article will be made available by the authors, without undue reservation.

## Author contributions

HK designed the research and wrote the manuscript. HK and FZ performed simulations and analyzed data. Both authors contributed to the article and approved the submitted version.
